# The RNA helicase Ddx5/p68 binds to hUpf3 and enhances NMD of Ddx17/p72 and Smg5 mRNA

**DOI:** 10.1093/nar/gkt538

**Published:** 2013-06-20

**Authors:** Verena Geißler, Simone Altmeyer, Benjamin Stein, Heike Uhlmann-Schiffler, Hans Stahl

**Affiliations:** Department of Medical Biochemistry and Molecular Biology, University of Saarland, Medical Center, Building 45, 66421 Homburg, Germany

## Abstract

Non-sense-mediated mRNA decay (NMD) is a mechanism of translation-dependent mRNA surveillance in eukaryotes: it degrades mRNAs with premature termination codons (PTCs) and contributes to cellular homeostasis by downregulating a number of physiologically important mRNAs. In the NMD pathway, Upf proteins, a set of conserved factors of which Upf1 is the central regulator, recruit decay enzymes to promote RNA cleavage. In mammals, the degradation of PTC-containing mRNAs is triggered by the exon–junction complex (EJC) through binding of its constituents Upf2 and Upf3 to Upf1. The complex formed eventually induces translational repression and recruitment of decay enzymes. Mechanisms by which physiological mRNAs are targeted by the NMD machinery in the absence of an EJC have been described but still are discussed controversially. Here, we report that the DEAD box proteins Ddx5/p68 and its paralog Ddx17/p72 also bind the Upf complex by physical interaction with Upf3, thereby interfering with the binding of EJC. By activating the NMD machinery, Ddx5 is shown to regulate the expression of its own, *Ddx17* and *Smg5* mRNAs. For NMD triggering, the adenosine triphosphate-binding activity of Ddx5 and the 3′-untranslated region of substrate mRNAs are essential.

## INTRODUCTION

Non-sense-mediated mRNA decay (NMD) is an mRNA quality control mechanism that protects eukaryotic cells from incomplete and potentially toxic proteins (1–4) and also regulates protein expression from a number of physiologically important mRNAs (5–10%) (5–11). Aberrant mRNAs with a premature translation termination codon (PTC) result from mutation or rearrangement of genomic DNA or defects in mRNA biogenesis. In mammals, the signal for their degradation is a translation-termination codon located at least 50–55 nt upstream of an exon–exon junction (1). Some physiological mRNAs have features, like upstream open reading frames (uORFs) or alternative splicing introducing non-sense codons or frameshifts that satisfy this constraint, and thus are targeted to this branch of NMD as well. According to the exon junction complex (EJC) model, EJC proteins Upf (upstream frame shifting) 2 and Upf3 (bound by MAGOH, Y14, and eIF4AIII) signal degradation of these mRNAs by binding to the SURF complex (consisting of Smg1, Smg9, Smg8, Upf1, eRF3 and eRF1) formed at the stalling ribosome (12–16). Other normal mRNAs have no exon–exon junction in such a position (6,7), and all their EJCs, which are deposited on the mRNA as a result of splicing in the nucleus, are removed from RNA by the translating ribosome during the first round of translation. A long 3′-untranslated region (3′-UTR) that would make translation termination events appear as premature, seem, to play a role for some in this mRNA class; triggering factor(s) are not defined (17–21). In any case, as with aberrant mRNAs, direct or indirect binding of Upf1 to the 3′-UTR might be envisaged as to result in a competition between Upf1 and cytoplasmic poly(A)-binding protein (PABP) for binding to the translation release factors eRF1 and eRF3 (19,22). And binding of the release factors to Upf1 at the terminating ribosome eventually stimulates its phosphorylation by the Smg1 kinase, translational repression and recruitment of decay enzymes (23–25). Conversely, binding of PABP to release factors is thought to preserve translational competence and transcript stability.

Ddx5 (p68) is a member of the DEAD box [a conserved motif named after its amino acid sequence (Asp-Glu-Ala-Asp)] subfamily of RNA helicases and plays a role in several RNA metabolic processes that require modulation of RNA secondary structures (26–29). Essentially, Ddx5 is a nuclear protein, which nevertheless shuttles between the nucleus and the cytoplasm (30). The biochemical activities of Ddx5 like RNA binding, adenosine triphosphate (ATP)-dependent RNA unwinding and RNA renaturation, are well characterized (28,29), although their role in specific functions is not well understood. As a multifunctional protein, it is involved in many processes in the cell. It can function as a transcriptional co-regulator with estrogen receptor-α, p53, MyoD and Runx2 [for review, see (31)], and a role in ribosome biogenesis, mRNA splicing and mRNA export has also been described (32–38). A high sequence identity exists in the central protein core with its paralog Ddx17 (but differing at N- and C-termini), of which two isoforms Ddx17_72_ (p72) and Ddx17_82_ (p82) are translated from the same *Ddx17* mRNA by use of different in-frame start codons (39,40). Ddx17 can interact with Ddx5 (41), and apparently most, but not all of their functions are redundant. The biological meaning of the Ddx17 isoforms is not known. Lacking specific Ddx17 reagents (antibodies), most studies have focused on Ddx5 or did not discern between both paralogs. Ddx5 expression is growth and developmentally regulated, and Ddx5 knockout mice are lethal around embryonic day 11.5 (42–45). Additionally, the differences in Ddx5 expression in a multitude of cancers indicate that Ddx5 may be important in cancer development (46–50).

In *Saccharomyces cerevisiae*, Dbp2p, the common homolog of Ddx5 and Ddx17, was described to interact with Upf1 and to function in the non-sense-mediated mRNA decay pathway (51). This and the fact that a cytoplasmic function of Ddx5 is still missing prompted us to search for a possible role of Ddx5 in the NMD of mammalian cells.

## MATERIALS AND METHODS

### DNA constructs

For pEGFP-Upf1, see (52); for Epstein–Barr virus-based knockdown–knockin constructs, pRTS-hygro and pRTS-pur, see (53); for luciferase NMD reporter constructs, see (54); for pCIneo-FLAG-Upf3B, see (55); and for pIRESneo-FLAG/HA-Ago2 see Addgene, Plasmid 10822. pCIneo-FLAG-Upf3B 1-270 and pCIneo-FLAG-Upf3B 270-470 were constructed from pCIneo-FLAG-Upf3B by deletion of respective sequences and pCMV-Ddx5-1-189-KT3 and pCMV-Ddx5-190-614-KT3 from pCMVp68-wt (32). For conditional gene expression, the Ddx5 coding sequence of pCMVp68-wt, pCMVp68DQAD and pCMVp68GNT (32) was amplified using respective primers with EcoRV-restriction sites, subcloned into pSfiExpress in front of an HA-tag and transferred into pRTS-hygro using SfiI restriction sites to yield pRTS-hygro-Ddx5-HA, pRTS-hygro-Ddx5_GNT_-HA and pRTS-hygro-Ddx5_DQAD_-HA. EGFP-3′-UTR-reporter constructs were obtained by inserting the EGFP sequence into pCIneo, and the 3′-UTR of the *Ddx17*, *Smg5, Ddx5* and *Tram1* gene, respectively, was cloned directly 3′ to the stop signal of EGFP and upstream of the SV40 late poly(A) signal using BspEI and NotI restriction sites. Used primer sequences are provided in Supplementary Table S1.

### Cell culture, cell transfection and exogenous gene expression

Cell lines were grown as described previously (32,40) with the exception of H1299 lung carcinoma cells that were maintained in Dulbecco’s modified Eagle’s medium with 10% fetal calf serum in 8% CO_2_. DNA and siRNA transfections were performed for 48–72 h using jetPEI (Peqlab) and Interferin (Peqlab), respectively, according to manufacturer’s protocol (for siRNA target sequences, see Supplementary Table S2 in Supplementary Information; AllStars Neg. Control siRNA from Qiagen was used as control). Conditional gene expression was accomplished in H1299 cells by transfection of pRTS-pur and a respective plasmid encoding the suitable HA-fusion protein in a pRTS-hygro background. Transfected cells were selected with puromycin (1 µg/ml) and hygromycin (200 µg/ml) for 5 days, and ectopic gene expression was induced by doxycycline (0.5 µg/ml) for 48 h. Cells were treated with wortmannin (10 µM), actinomycin D (5 µg/ml) and cycloheximide (100 µg/ml) solved in dimethyl sulfoxide (DMSO) 40 h after transfection where indicated. For EGFP-3′-UTR-reporter assays, HeLa cells were transfected with respective EGFP-3′-UTR-reporter constructs and splitted thereafter (24 h) to establish individual cultures for siRNA transfection as described previously (54).

### Antibodies

Rabbit anti-Upf1 (sc-48802), goat anti-Upf2 (sc-20227), rabbit anti-Upf3 (sc-48800), goat anti-eIF4G (sc-9601), mouse anti-PABPC1 (sc-166027), goat anti-PABPN1 (sc-33007), mouse anti-CBP80 (sc-271304), goat anti-Smg5 (sc-50980), mouse anti-MAGOH (sc-56724) and goat anti-eIF4E (sc-6968) antibodies were from Santa Cruz. Mouse anti-tubulin (#05-829) was from Millipore, mouse anti-GFP (MAB3580) was from Upstate, rat anti-HA (clone 3F10) was from Roche, rabbit anti-eRF3 (ab49878) was from Abcam and mouse anti-FLAG M2 (F3165) was from Sigma. For monoclonal mouse antibody C10 (raised against the carboxy-terminal 15 amino acids) and polyclonal rabbit anti-human Ddx17 (Ddx17_72_/Ddx17_82_) antibody (raised against a Ddx17 deletion mutant; consisting of amino acids 437–650 and recognizing both paralogs) (32,40), for monoclonal antibodies PAb101 (56), and for KT3 (57). Horseradish peroxidase-conjugated antibodies goat anti-mouse (A4416) and goat anti-rabbit (A0545) were from Sigma, goat anti-rabbit (12-348) was from Millipore and donkey anti-goat (sc-2020) was from Santa Cruz. The fluorescence-conjugated secondary antibody goat anti-mouse-Alexa-Flour 568 was from Invitrogen.

### Co-immunoprecipitation assay

All steps were performed at 4°C. HeLa cells were harvested in phosphate-buffered saline by centrifugation, resuspended in lysis buffer (20 mM HEPES–NaOH, pH 7.5, 10 mM NaCl, 2 mM MgCl_2_, 1 mM EGTA, 0.35% Nonidet P-40 and 0.2% Na-desoxycholate, pH 8.6) supplemented with 1 mM PMSF, 2 mM NaVO_3_ and Complete Protease Inhibitor ethylenediaminetetraacetic acid free (Roche). Cell lysates were centrifuged at 14 000*g* for 10 min, pre-cleared with protein A-sepharose beads and incubated over night with protein A-sepharose-bound antibodies in the presence or absence of RNase A (Novagen; RNA digestion was controlled by agarose gel electrophoresis). After extensive washing of the immunopellets with washing buffer (lysis buffer containing 1% Nonidet P-40), bound proteins were analyzed by western blotting as described previously (32). For RNA immunoprecipitation (RIP), HeLa cell extracts were prepared and processed for immunoprecipitation with monoclonal anti-Ddx5 antibody C10 as described earlier in the text but in the presence of RiboLock RNase Inhibitor (Fermentas) in all steps. The immunopellet was washed six times with NT2 buffer (50 mM Tris, pH 7.4; 150 mM NaCl, 1 mM MgCl_2_ and 0.05% Nonidet P-40) and peptide eluted for 1 h in 200 µl NT2 buffer containing 2 mg of a peptide representing the C10 epitope. RNA was purified from the eluent by proteinase K digestion, phenol–chloroform–RNA extraction and ethanol precipitation. In a negative control experiment, the SV40 large tumor antigen-specific monoclonal antibody PAb101 was used. Pull-down assays were performed by incubation of the respective bait-protein with antibody-loaded Protein A-sepharose beads in the presence of RNase A over night. After washing the beads with washing buffer, cell extracts containing the respective prey protein were added, and after an additional incubation period of 4 h, the immunopellets were processed as described earlier in the text.

### Immunofluorescence

For immunofluorescence analysis, HeLa cells were transfected with the indicated plasmids for 48 h by using nanojuice (Merck) according to the user manual and further processed as described previously (32).

### mRNA isolation and quantification

Total RNA was extracted from cells using the RNeasy Mini Kit (Qiagen) according to the manufacturer’s protocol. After DNase treatment, 1 µg of total RNA was converted to cDNA using anchored-oligo(dT)_18_ or random hexamer primers and the Transcriptor High Fidelity cDNA Synthesis Kit (Roche). Quantitative real-time reverse transcriptase–polymerase chain reaction (RT–PCR) was performed with TAMRA-labeled TaqMan gene expression assays specific for Ddx17 (HS 00978019_m1), 18SrRNA (HS99999901_s1), Smg5 (HS 00383399_m1) or GAPDH (HS02758991_g1) cDNA by using the 7500 Fast Real-Time PCR System from Applied Biosystems. The EGFP-specific TaqMan probe and primer set was as described (58). mRNA concentrations were normalized to 18 S rRNA or GAPDH transcript levels as indicated, using the comparative ΔCT method (59). mRNA half-lives (t_1/2_) were calculated from the slope of the trend lines. Statistical analysis was performed using the two-tailed Student’s *t*-test. For semi-quantitative mRNA analysis, gene-specific or EGFP-specific (for 3′-UTR-reporter constructs) primers (Supplementary Table S3) were used for cDNA amplification in combination with the Kappa Robust PCR Kit according to the manufacturer’s protocol (Roche) and as described previously (32). PCR products were analyzed by 1% agarose gel electrophoresis and EtBr staining.

### NMD-reporter assays

NMD-reporter assays were performed as described (54), except that Interferin (Peqlab) was used instead of Oligofectamine (Invitrogen) and jetPEI (Peqlab) instead of CaPO_4_ reagent for RNA and DNA transfection, respectively. Total RNA was extracted from cells using the RNeasy Mini Kit (Qiagen) according to the manufacturer’s protocol. After DNase treatment, 1 µg of total RNA was converted to cDNA using random hexamer primers and the Transcriptor High Fidelity cDNA Synthesis Kit (Roche). Specific primers for firefly or renilla cDNA (Supplementary Table S3) were used for amplification. PCR products were analyzed by 1% agarose gel electrophoresis and EthBr staining.

## RESULTS

### Ddx5 interacts with human NMD key factors and is associated with mRNPs

**Figure 1.**
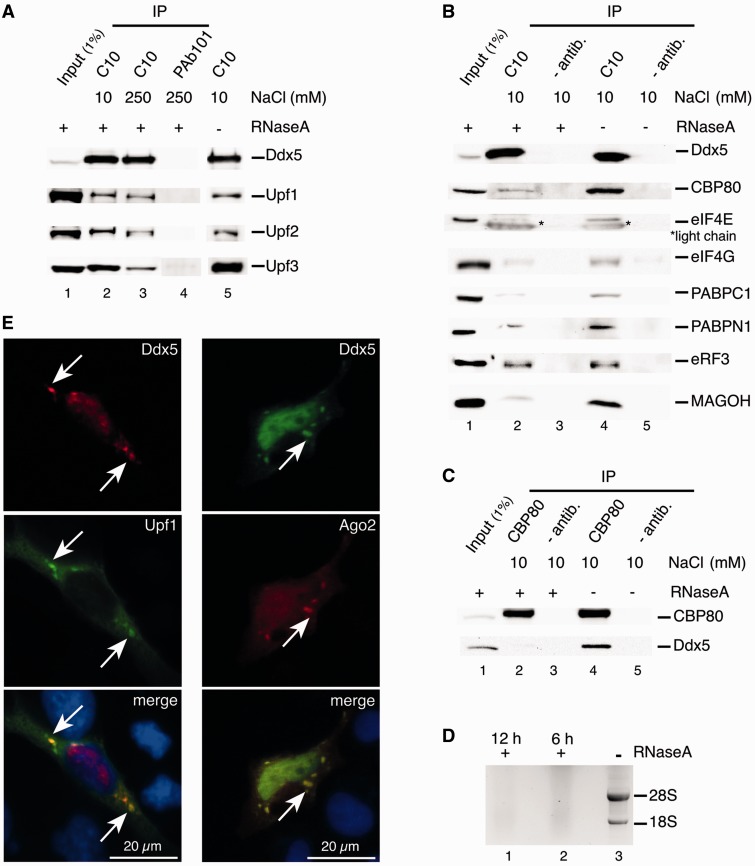
Interaction of Ddx5 with human NMD key factors and mRNPs. (**A**) Co-IP of human Upf1, Upf2 and Upf3 with Ddx5. HeLa cell lysates were immunopreciptated with an anti-Ddx5-antibody (C10) or a control antibody (PAb101) in the presence (+) or absence (−) of RNase A at different NaCl concentrations. Ddx5, Upf1, Upf2 and Upf3 were detected with respective antibodies by western blotting. (**B**) Co-IP of mRNP components with Ddx5. HeLa cells were subjected to IP using an anti-Ddx5 antibody (C10) or no antibody as a control (− antib.) in the presence (+) or absence (−) of RNase A. Immunopurified proteins were analyzed by western blotting with respective antibodies. Asterisks mark mouse IgG light chains stained by the secondary antibody because of their cross-reactivity. Input analysis without RNase A essentially gave the same result as that with RNase A and is not shown. (**C**) Co-IP of Ddx5 with CBP80. HeLa cells were subjected to IP using an anti-CBP80 antibody or no antibody (− antib.) in the presence (+) or absence (−) of RNase A. Immunoprecipitated proteins were analyzed by western blotting with respective antibodies. (**D**) Control of RNase A digestion of cell extracts used after indicated times by agarose gel electrophoresis. We show the EthBr staining of the gel with marked 28 S and 18 S rRNA. (**E**) Co-localization of Ddx5 and Upf1 (left panel) and Ddx5 and Ago2 (right panel) in HeLa cells. HeLa cells co-transfected with vectors encoding Ddx5-KT3 (red) and Upf1-GFP (green) or Ddx5-KT3 (green) and Ago2-HA (red) were analyzed by immunofluorescence microscopy. Nuclei were stained with DAPI (blue). White arrows indicate co-localization of Ddx5 with Upf1 and Ago2, respectively, in cytoplasmic granules.

We analyzed possible Ddx5 interactions with components of the NMD machinery and performed immunopurification (IP) assays in HeLa cell lysates, using a monoclonal anti-mouse antibody, raised against the C-terminus of Ddx5 (C10; Figure 1A). A robust signal for the three human (h) key NMD factors, hUpf1, hUpf2 and hUpf3 was observed with the latter clearly predominating quantitatively (the Upf3 antibody used in the western-blotting experiment binds to both paralogs, hUpf3A and hUpf3B; 60). IP of the Upfs was resistant to RNase A treatment (for efficiency of RNase treatment, see Figure 1D), and although RNA may be required for formation of the underlying protein–protein interactions, it seems dispensable for their stability. When performed at higher ionic strength (250 mM NaCl) some hUpf3 was removed, yet bound Upfs now appeared at about stoichiometric level indicating a more stable Ddx5 interaction with a preformed hUpf1-hUpf2-hUpf3 complex [with Upf2 bridging Upf1 and Upf3; (16,17)].

Functional Upf1-Upf2-Ufp3 complexes are integral components of messenger ribonucleoproteins (mRNPs), whereby the overall protein composition of an mRNP depends on its function, apparent, e.g. through the replacement of nuclear cap-binding protein CBP80 by the cytoplasmic cap-binding protein eIF4E (eukaryotic translation initiation factor 4E) after the first (pioneer) round of translation. Further mRNP components are the Poly(A)-binding protein C1 (PABPC1), which replaces PABPN1 in steady-state mRNAs (61) and can bind either to the eukaryotic initiation factor 4G (eIF4G) to enhance translation or to eukaryotic release factor 3 (eRF3) for translation termination [reviewed in (62)]. Detailed protein analysis of the Ddx5-specific immunoprecipitates revealed some of these typical mRNP components, like CBP80, eIF4E, eIF4G, MAGOH, PABPC1 and PABPN1, bound to Ddx5 in an RNase-sensitive manner, suggesting an indirect, RNA-mediated interaction with Ddx5. An exception was seen with eRF3, which bound to the immunocomplex in RNase A-resistant manner (Figure 1B; for analysis of bound mRNA, see later in the text). This may be explained by the known physical interaction of Upf1 with eRF3, possibly during stop codon recognition in the state of translation termination (63). Notably, most Ddx5 were associated with mRNPs involved in their pioneer round of translation as deduced from the ratios of bound CBP80 and PABPN1 compared with their steady-state mRNA-specific counterparts (64), as well as from the presence of MAGOH, a core protein of EJCs removed from normal mRNAs by bypassing ribosomes. We are aware, however, that the exchange of CBP80 for eIF4E is a translation-independent process (61); thus, some CBP80 in Ddx5-specific IPs may be derived from steady-state mRNPs and the small amount of detected eIF4E, on the other hand, from mRNPs involved in the first round of translation (65). Co-imunoprecipitates obtained with CBP80 antibodies also contained Ddx5 in an RNase A-sensitive manner, although only a small fraction of cellular Ddx5 was involved (Figure 1C).

The interaction with the hUpf complex points to a potential role of Ddx5 in NMD processes, in the course of which mRNP complexes may pass through processing bodies [P-bodies; (66)]. In fact, fluorescent micrographs of HeLa cells, co-expressing a GFP fusion of hUpf1 and KT3 epitope-tagged Ddx5 (labeled by KT3 antibody), disclosed co-localization of both proteins in cytoplasmic granules besides their expected accumulation in the nucleus and cytoplasm, respectively (Figure 1E). The identification of those granules as P-bodies was realized by co-localization of Ddx5 and Ago2, a bona fide P-body marker, in these structures (67–69).

### Ddx5 physically interacts with hUpf3B

**Figure 2.**
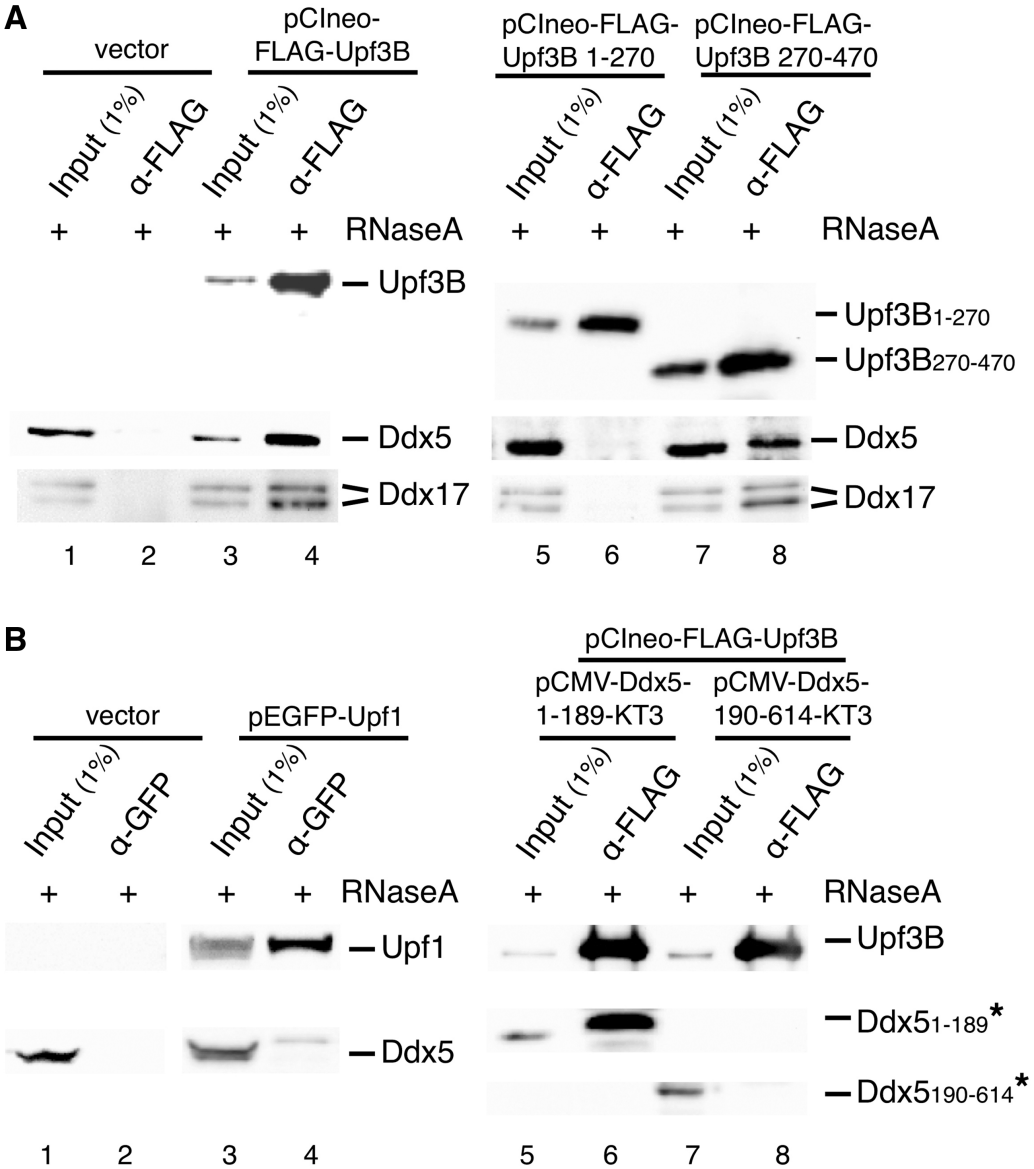
Physical interaction of Ddx5 and hUpf3B. (**A**) Binding of Ddx5 to the C-terminal part of Upf3B. HeLa cells were transfected with plasmids encoding FLAG-tagged Upf3B or one of the Upf3B deletion mutants (FLAG-Upf3B_1–270_ or FLAG-Upf3B_270–470_) or with an empty vector (vector) for 48 h. Cells were lysed in the presence of RNase A, immunoprecipitated with anti-FLAG antibodies and analyzed for indicated proteins by western blotting. (**B**) Binding of Upf3B to the N-terminal part of Ddx5. Left panel, HeLa cells were transfected with GFP-Upf1 for 48 h, and cell lysates were immunoprecipitated with anti-GFP antibodies followed by western blot analysis of indicated proteins. Right panel, HeLa cells were transfected with plasmids encoding FLAG-tagged Upf3B and one of the KT3-tagged Ddx5 deletion mutants (Ddx5-KT3_1–189_ or Ddx5-KT3_190–614_). Pull-down assays were performed with anti-FLAG-antibodies and Ddx5-KT3 deletion mutants as the prey-proteins (labeled by asterisks) in the presence of RNase A followed by western blot analysis with anti-Upf3B and anti-KT3 antibodies.

To determine which of the Upfs binds to Ddx5, we expressed FLAG-tagged hUpf3B or EGFP-tagged hUpf1 in HeLa cells and investigated possible interactions by Co-IP (Figure 2A and B). The data show that Upf3B preferentially binds to Ddx5 (and also Ddx17; Figure 2A, lanes 1–4), whereas Upf1 did not (Figure 2B, lanes 1–4). The EGFP-tag is reported not to disturb complexing of the Upf proteins with each other (52), which explains some Ddx5 co-immunoprecipitated (indirectly) with EGFP-hUpf1 (Figure 2B; note that the fraction of Ddx5 bound to complexes containing endogenous Upf1 is not precipitated by the α-GFP-antibodies). Expressing the deletion mutant Upf3B_1__–__270_ still able to interact with hUpf2 (15,70), no Ddx5 was co-precipitated, thus excluding also any physical contact of Ddx5 and hUpf2. However, the non-hUpf2-binding mutant hUpf3B_270__–__470_ efficiently bound Ddx5, indicating that the Ddx5:Upf3 interaction proceeds via the C-terminal half of hUpf3B with hUpf1 and hUpf2 being dispensable [Figure 2A, lanes 5–8; (16)]. We note, however, that binding of hUpf2 to hUpf3B is not impeded by the Ddx5:Upf3 complex formation (Figure 1). To narrow down the region on Ddx5 responsible for hUpf3B interaction, pull-down assays were performed using purified recombinant FLAG-tagged hUpf3B and KT3-tagged Ddx5 deletion variants, expressed in HeLa cells. Co-precipitation of Ddx5_1__–__189_ and Upf3B was observed in these experiments, whereas Ddx5_190__–__614_ failed to interact with Upf3B. Thus, we conclude that amino acids 1–189 of Ddx5, containing the ATP-binding motif (Walker A), are sufficient for Upf3 binding (Figure 2B, lanes 5–8). However, until binding is shown to occur using purified proteins, one will not know, however, if the interaction is direct.

### Ddx5 regulates the expression of Ddx17 by NMD induction

**Figure 3.**
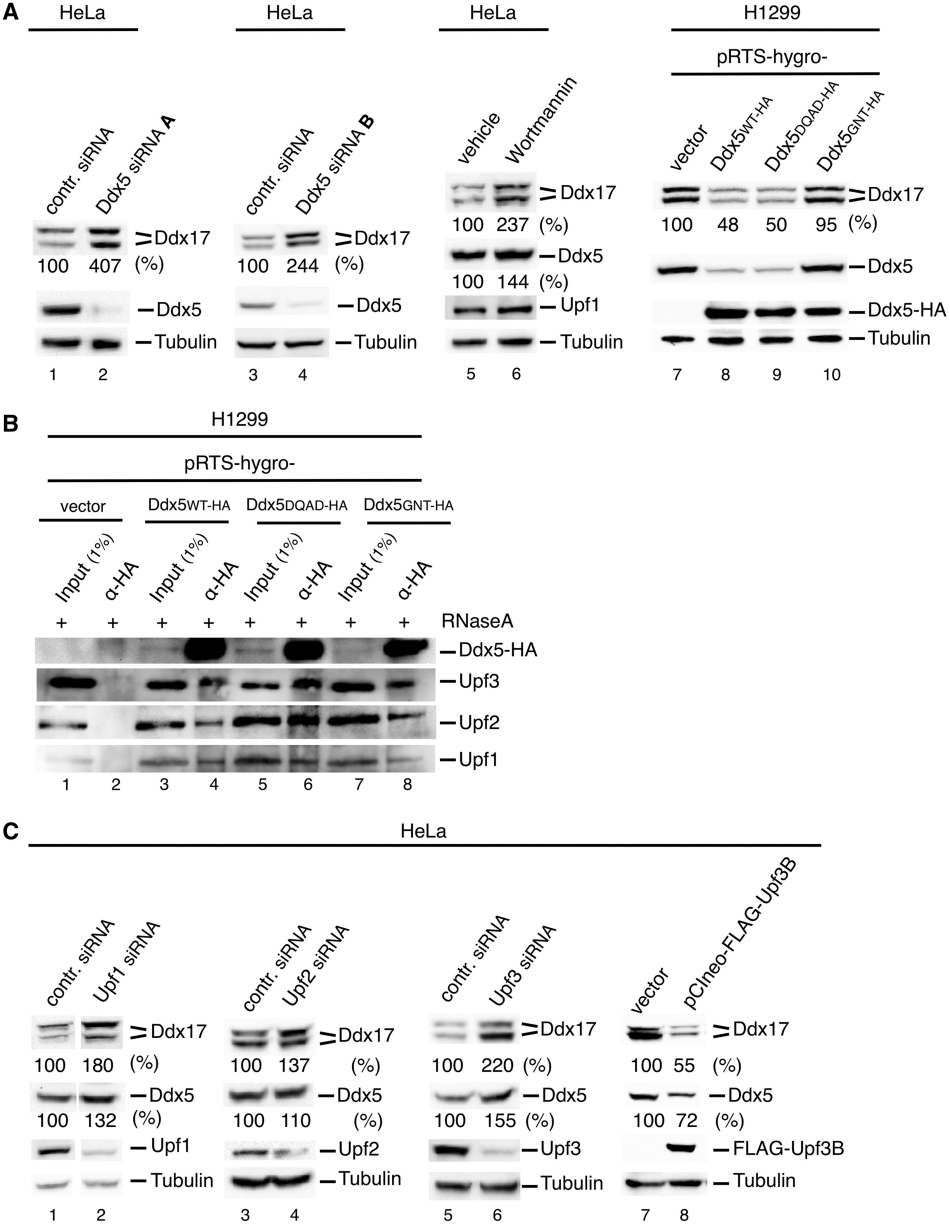
Regulation of Ddx17 expression by Ddx5-dependent NMD. (**A**) Effects of Ddx5 and Wortmannin. Lanes 1–6: HeLa cells were transfected with indicated siRNA or were treated with Wortmannin (10 µM in 0.5% DMSO; lane 6) or the vehicle only (lane 5) and harvested for western blot analysis 48 h thereafter. Lanes 7–10: H1299 cells were transfected for conditional gene expression with pRTS-pur and indicated pRTS-hygro-plasmids and selected with puromycin (1 µg/ml) and hygromycin (200 µg/ml) for 5 days followed by induction of protein expression by doxycycline (0.5 µg/ml) for 48 h and western blot analysis. An anti-HA antibody is used to detect Ddx5-HA and antibody C10 to detect endogenous Ddx5 (C10 binds to C-terminus of Ddx5 and does not recognize the C-tagged protein). (**B**) Binding of Ddx5 mutants to Upf3, Upf2 and Upf1. H1299 cells were transfected with plasmids as described in (A), and cell lysates were immunoprecipitated with anti-HA antibodies followed by western blot analysis of indicated proteins. (**C**) Effects of NMD factors Upf1, Upf2 and Upf3. HeLa cells were transfected with indicated siRNAs or with a FLAG-Upf3B-encoding plasmid. Cells were harvested 48 h thereafter and analyzed by western blotting of indicated proteins. In (A) and (C), tubulin was used as a loading control, and Ddx17 and Ddx5 levels are given as % of respective controls.

To activate the NMD process, Upf3 (via Upf2) either links the EJC to (15,16) or directly interacts with Upf1 (22,71); thus, the detected Ddx5/Ddx17-Upf3 interaction prompted us to investigate the notion that these DEAD box proteins are involved in NMD processes as well. As it is known that Ddx5 controls the expression of itself and its paralog Ddx17 (32), we investigated this phenomenon for NMD-specific properties. First, the ability of two Ddx5-specific siRNAs (Ddx5 siRNA A and Ddx5 siRNA B) to upregulate endogenous Ddx17 expression in HeLa cells (including both isoforms, which we refer to as Ddx17_72_ and Ddx17_82_) was confirmed by western blot analysis [Figure 3A, lanes 1–4; see also (32)]. As both siRNAs showed similar effect, only Ddx5 siRNA A was used in the following experiments. Next, H1299 human lung carcinoma cells were stably transfected with pRTS-1 constructs, conditionally expressing HA-tagged Ddx5 wild-type (WT) or Ddx5 mutants in a doxycycline-dependent manner (53). Exogenous gene expression was activated for 48 h resulting in high cellular Ddx5 expression levels overriding that of the endogenous protein (at least 3-fold, data not shown). Our results indicate that both isoforms of Ddx17 are downregulated by its paralog Ddx5, and that the expression of exogenous also results in a suppression of endogenous Ddx5, pin-pointing an additional autoregulatory control (Figure 3A, lanes 7 and 8). Both effects depended on the ATP binding, but not ATP hydrolysis/helicase activity of Ddx5 as revealed by overexpression of mutant Ddx5-GNT (mutated in the ATP-binding/Walker A motif) and Ddx5-DQAD (mutated in the DEAD box /Walker B motif; Figure 3A, lanes 7–10). Both mutants have no ATPase and RNA helicase activity, but (like the wild-type) bound equally well to the Upf3-Upf2-Upf1 complex (Figure 3B). However, that mutant Ddx5-GNT additionally lacks ATP-binding activity (32), which may affect conformational changes of Ddx5 in the induction of the NMD process (72–74). A decrease in the cellular Ddx17 level caused by Ddx17 siRNA treatment, on the other hand, had no substantial effect on Ddx5 expression (32), thus indicating that the cross-regulatory relationship between Ddx5 and Ddx17 is unidirectional.

We wondered whether the expression control by Ddx5 is explained exclusively by variations in splicing efficiency as discussed previously (32), or may as well be attributed to a cytoplasmic control like, e.g. NMD activity. To test a possible influence of the NMD process on Ddx5 and Ddx17 expression, we analyzed their protein level in HeLa cells after Upf1-inactivation by Wortmannin, which blocks the phosphorylation of Upf1 by the phosphatidyl-inositol-3-related kinase Smg1 (54,75). As can be seen in Figure 3A, Wortmannin treatment increased the expression of Ddx5 (1.6-fold) and Ddx17 (>2-fold; Figure 3A, lanes 5 and 6). In addition, knockdown of hUpf1, hUpf2 and hUpf3 by RNAi and overexpression of FLAG-tagged Upf3B also showed negative or positive effects, respectively, on cellular Ddx5 and Ddx17 levels, proposing a regulation by the NMD machinery (Figure 3C). Interestingly, the influence on Ddx5 is less pronounced most probably because the autoregulation of Ddx5 is not as strong as the downregulation of Ddx17 in HeLa cells.

**Figure 4.**
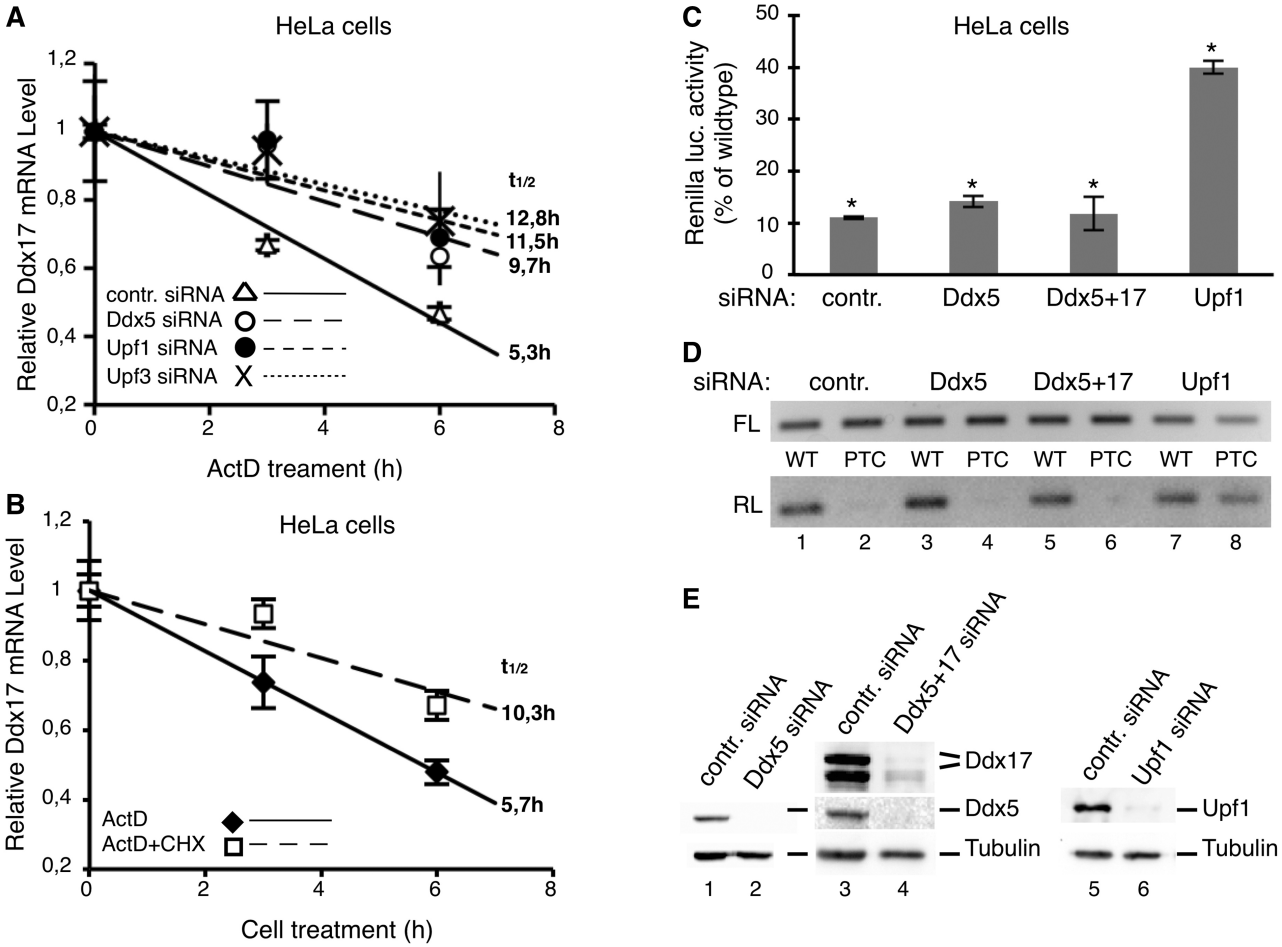
Influence of the Ddx5-dependent NMD on the stability of Ddx17 mRNA but not on the expression of a PTC-containing reporter gene. (**A** and **B**) Half-life of Ddx17 mRNA in dependence on Ddx5, Upf1, Upf3 and ongoing translation. (A) HeLa cells were transfected with indicated siRNAs and 48 h thereafter treated with actinomycin D (ActD; 5 µg/ml;) (B), untransfected HeLa cells were treated with actinomycin D or actinomycin D plus cycloheximide (CHX; 100 µg/ml) for indicated times. Thereafter, total RNA was isolated in A and B, and Ddx17 mRNA levels were determined by real-time RT–PCR normalized to 18SrRNA (mean ± SD; *n* = 2–3, t_1/2_ = calculated half life). (**C** and **D**) No effect of Ddx5 on the NMD of a PTC-containing reporter. HeLa cells were transfected with a firefly-luciferase plus a renilla-luciferase expression construct (containing the renilla luciferase/β-globin fusion cDNA sequence with (PTC) or without (WT) a PTC at codon 39 of the β-globin open reading frame) and split thereafter for knockdown of Ddx5 (Ddx5_si_), Ddx5/Ddx17 (Ddx5+17_si_) and Upf1 (Upf1_si_), respectively. Lysates of the cell populations were prepared 48 h thereafter. (**C**) Analysis of renilla- (normalized to firefly-) luciferase activity by luminometry (mean ± SD; *n* = 3; *P* < 0.0005). The renilla luciferase activity expressed from the PTC-containing reporter is given in relation to that from the wild-type (no PTC) construct. (**D**) Analysis of renilla-luciferase mRNA (RL) by RT–PCR normalized to firefly-luciferase mRNA (FL). (**E**) Efficiencies of the respective protein knockdowns were checked by western blotting (with tubulin as a loading control).

We also performed RNA half-life analysis to check whether increased steady-state levels of Ddx17 in response to Ddx5 and Upf1, Upf2 and Upf3 knockdown result from increased mRNA synthesis. This analysis detected a 2-fold increase in mRNA stability in dependence on Ddx5, as well as hUpf1 or hUpf3 RNAi knockdown, and in line with the results aforementioned, points to a role of these proteins in an NMD-driven degradation of *Ddx17* mRNA (Figure 4A). Furthermore, consistent with the concept that ongoing translation is essential for NMD (76), the accelerated decay of the *Ddx17* transcript was also blocked by cycloheximide (Figure 4B).

### Ddx5 does not affect PTC-mediated NMD

NMD of aberrant transcripts can be provoked by a premature translation termination codon, and we asked whether Ddx5 is involved also in this process. Thus, we assessed the effect of Ddx5 knockdown on the expression of a PTC-containing transcript by using a chemiluminescence-based reporter system (54). The used reporter constructs contain an in-frame renilla luciferase/β-globin fusion gene with (PTC) or without (WT) a non-sense mutation at codon 39 of the β-globin open reading frame. A co-transfected firefly luciferase construct was used for normalization. Because Ddx17 also interacts with hUpf3B (Figure 2A), and thus may have a similar function as Ddx5, we also used an siRNA, which simultaneously knocks down both paralogs. However, in contrast to hUpf1, these experiments revealed no influence of Ddx5 and/or Ddx17 on the expression of the renilla luciferase/β-globin fusion gene irrespective of the presence or absence of PTC both on mRNA (Figure 4D) and on protein level (Figure 4C).

### Ddx5 causes NMD of Smg5

**Figure 5.**
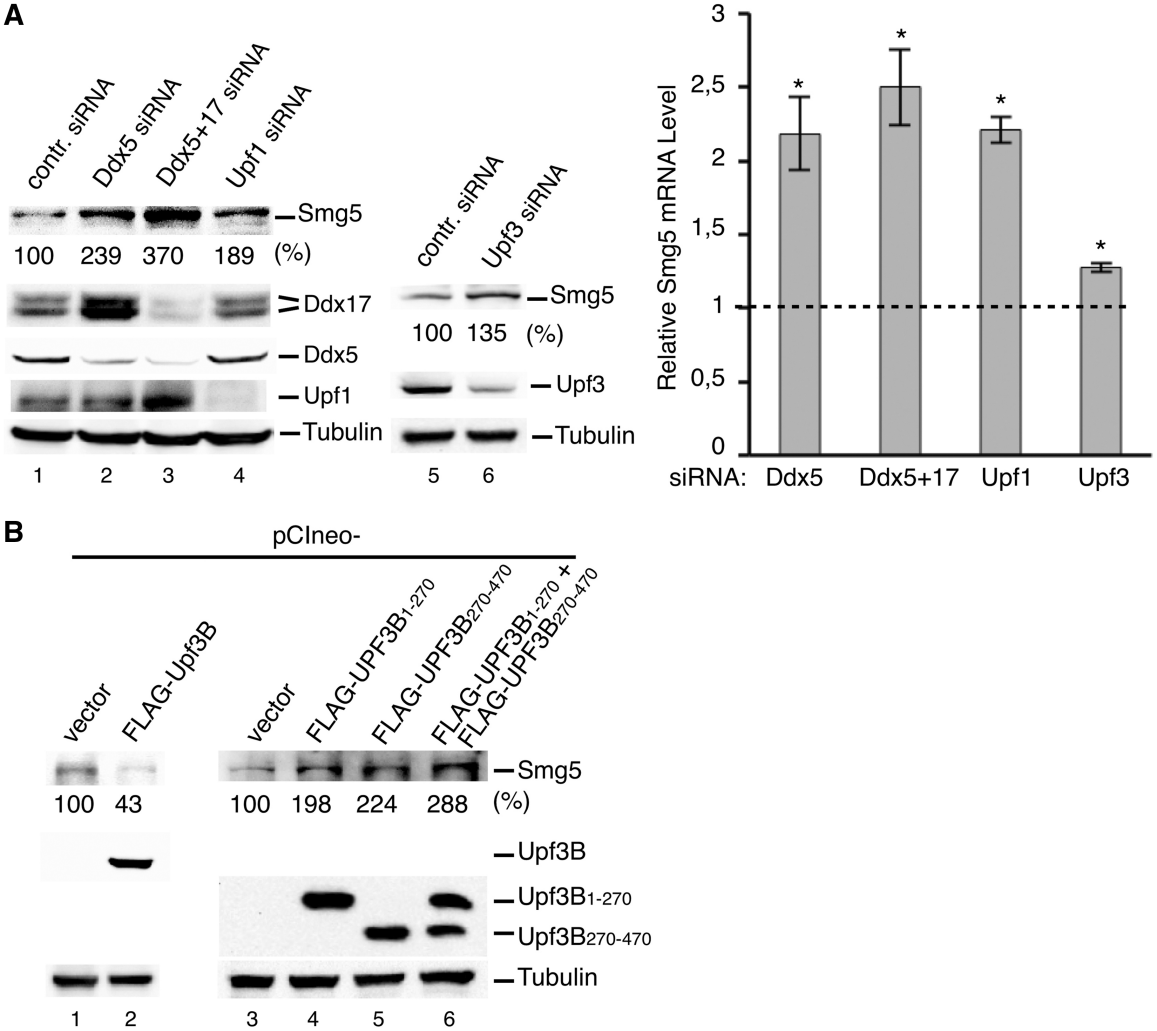
Control of Smg5 expression by Ddx5, hUpf1 and hUpf3. (**A**) Cellular Smg5 protein and mRNA levels after knockdown of Ddx5, Ddx5/Ddx17, hUpf1 or hUpf3. Forty-eight hours after transfection with indicated siRNAs, HeLa cells were analyzed by western blotting or real-time RT–PCR for Smg5 protein (with tubulin as loading control, left panel) and mRNA (normalized to *GAPDH* mRNA levels and expressed as fold change; mean ± SD; *n* = 3; *P < 0.05; right panel) in comparison with the control (dashed line). (**B**) Cellular Smg5 levels in dependence on Upf3. HeLa cells were transfected with a plasmid encoding FLAG-Upf3B or deletion mutants thereof (FLAG-Upf3B_1–270_ and FLAG-Upf3B_270–470_, respectively) for 48 h and analyzed by western blotting. Smg5 levels are given as % of control siRNA or empty vector transfected cells in (A) and (B).

Our observation that Ddx5 regulates the expression of *Ddx17* in an Upf1, Upf2 and Upf3-dependent manner suggests that it also marks other physiological mRNAs for the NMD machinery. According to a genome-wide search, one common property of physiological mRNAs controlled by the NMD machinery is a long 3′-UTR, which is present in the *Ddx17* transcript (2455 nt) and, e.g. also in the *Smg5* mRNA (1342 nt). The latter itself is an NMD factor and shown recently to be regulated in a hUpf1 and 3′-UTR-dependent manner (7.19, 77,78). We surmised that co-translational surveillance of this physiological mRNA also depends on Ddx5 and tested this hypothesis by comparing the effects of either hUpf1 or Ddx5 siRNA knockdown on Smg5 expression in HeLa cells. As shown in Figure 5A, downregulation of either one led to a distinct increase in cellular levels of *Smg5* mRNA and protein. In addition, when Ddx5 and Ddx17 were knocked down simultaneously, *Smg5* expression was even more enhanced, indicating similar activities of both paralogs. The Ddx5-dependent NMD of Smg5 seems comparatively impervious to Upf3 depletion by the RNAi approach [Figure 5A; see also (18)]. We found, however, that expression of mutants hUpf3B_1__–__270_ (not binding Ddx5) and/or hUpf3B_270__–__470_ (not binding hUpf2) led to ∼2 - to 3-fold increase in cellular Smg5 levels (Figure 5B, lanes 3–6), whereas overexpression of the wild-type, on the other hand, results in a clear *Smg5* downregulation (∼60%; Figure 5B, lanes 1 and 2).

### Ddx5 directs NMD of Ddx5, Ddx17 and Smg5 via their 3′-UTRs and preferentially binds to these mRNAs *in vivo*

**Figure 6.**
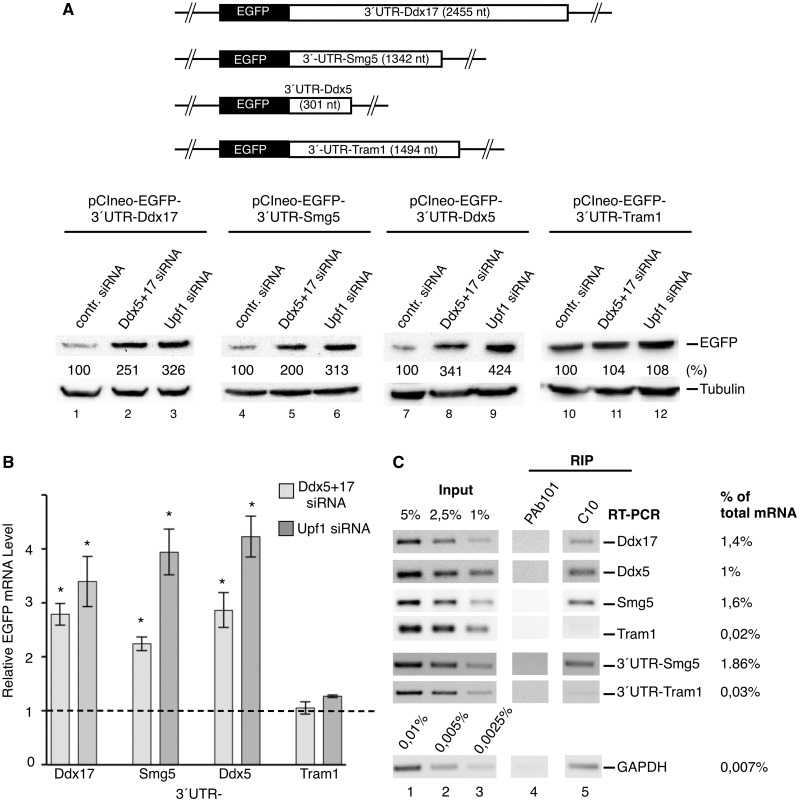
Role of the 3′-UTR in Ddx5 controlled NMD and preferred binding of Ddx5 to NMD substrates *in vivo*. (**A** and **B**) NMD of a reporter gene in dependence on individual 3′-UTRs and Ddx5/Ddx17. (A) HeLa cells were transfected with expression constructs encoding the EGFP-cDNA fused with its 3′-end to the 3′-UTR of indicated mRNAs. After knockdown of paralogs Ddx5/Ddx17 (Ddx5+17 siRNA) or hUpf1 (Upf1 siRNA), cells were analyzed by western blotting using anti-EGFP antibodies (with tubulin as a loading control). A schematic representation of used reporter genes with the length of indicated 3′-UTRs (in nucleotides) is given on top. (B) In a parallel experiment, cellular *EGFP* mRNA levels were determined by real-time RT–PCR normalized to *GAPDH* mRNA levels and expressed as fold change in comparison with the control (dashed line; mean ± SD; *n* = 2–3; *P < 0.005). (**C**) *In vivo* mRNA binding of Ddx5. A semi-quantitative RT–PCR analysis of endogenous or plasmid driven (3′-UTR-Smg5 and 3′-UTR –Tram1; indicated to the right) transcripts obtained by RIP from HeLa cells with antibody C10 (PAb101 used as a control antibody) was performed, and mRNA levels in immunoprecipitates are given as % of total. An agarose gel-electrophoretic analysis of RT–PCR-products obtained from immunoprecipitated RNA (RIP) and three concentrations of total RNA (Input) is shown.

To prove that the 3′-UTR of respective mRNA is indeed the regulating element responsible for mRNA degradation, the EGFP-reporter DNA sequence was cloned in front of the *Ddx17*, *Smg5, Ddx5* and *Tram1* (translocating chain-associated membrane protein 1) 3′-UTR sequence, respectively, and the influence of Ddx5/Ddx17 and hUpf1 on the expression of these constructs was compared with each other. The *Tram1*-3′-UTR construct was used as a negative control because it was shown before not to stimulate the NMD process in spite of its length [1494 nt; (19)]. As with the authentic mRNAs, the 3′-UTR sequences led to the stabilization the respective transcripts (Figure 6B) and a strong increase in the expression of the reporter by a factor of two to four after knockdown of Ddx5/Ddx17 or hUpf1 (Figure 6A). Notably, the 3′-UTR of *Tram1* showed no such effect in this assay (Figure 6A and B), indicating that Ddx5 induces the NMD of physiological mRNA via interaction with specific 3′-UTR sequences or structures (Figure 6A and B). Moreover, these data also propose that *Ddx5* mRNA is an NMD substrate.

To verify that Ddx5 associates with those mRNAs it regulates *in vivo*, we performed RNA immunopurification (RIP) assays in HeLa cell lysates, using the anti-Ddx5 antibody C10 in combination with peptide elution (Figure 6C). As determined by semi-quantitative RT–PCR analysis of precipitated RNAs, 1.4, 1 or 1.6% of total cellular *Ddx17-*, *Ddx5*- and *Smg5-* mRNA, respectively, specifically were bound to Ddx5 in comparison with only 0.02% of *Tram1*- and 0.007% of GAPDH–mRNA. In addition, we also found that Ddx5 specifically binds to the 3′-UTR-Smg5 reporter mRNA (1.86%) but not to that of the 3′-UTR-Tram1 construct (0.03%; Figure 6C). However, when we tested the PTC- versus the WT-renilla luciferase/β-globin construct (Figure 4C and D) in the RIP assay, we detected no difference in Ddx5 affinity (data not shown). Taken together, these results confirm a function of Ddx5 in the regulation of *Ddx17-, Smg5-* and also *Ddx5*- mRNA that seem to be recognized as NMD substrates by Ddx5 binding to their 3′-UTRs. Interestingly, Upf1 has also been shown recently to preferentially associate with transcripts containing 3′-UTRs (including *Smg5*) known to elicit NMD (79); in the light of our data, this could be an indirect interaction mediated by Ddx5 or analogous factors.

## DISCUSSION

In the degradation of PTC-containing mRNAs, Upf3, in concert with Upf2, is the link, which redirects the signal for mRNA degradation from the EJC to the NMD machinery. Up to now, no other factor was shown to be capable of substituting for EJC in alternative branches of the NMD pathway. Other known NMD activators, like Staufen1, by-pass this chain-reaction by binding directly to Upf1 (4). We show here that Ddx5 and hUpf3B interact physically. This interaction is limited to the N- and C-terminal part of Ddx5 and hUpf3, respectively, and, like the Upf3-EJC interaction, does not interfere with the binding of Upf3 to Upf2. The C-terminus of Upf3B has also been shown to interact with a composite binding surface of EJC, which, however, seems not to be compatible with its binding to Ddx5 as deduced from the absence of MAGOH, one of the EJC core proteins, in the Ddx5-specific immunoprecipitates after RNA digestion. Indeed, we have found that Ddx5 does not influence the expression of a PTC-containing reporter, which implies mutually exclusive Upf3B-binding sites for Ddx5 and EJC that specify alternative branches of the NMD.

Consequently, we have analyzed the ability of Ddx5 to accelerate the NMD of physiological transcripts, given that it interacts with them in a proper way. Indeed, we have disclosed three target mRNAs, *Ddx5*, *Ddx17* and *Smg5* mRNA, regulated by Ddx5 in cooperation with NMD factors Upf1, Upf2 and Upf3. Previous results suggested that the Ddx5-mediated negative expression control of its own and of Ddx17 functions in part at the level of splicing as the nuclear pool of their partially spliced pre-mRNAs decreased at low Ddx5, whereas that of the mature mRNAs increased (32). We add to this a distinct (2-fold) increase in *Ddx17* mRNA half-life observed in dependence on Ddx5 knockdown. This and the dependence of the expression control on Upf1, Upf2 and Upf3 and on Wortmannin point to the degradation of the *Ddx5* and *Ddx17* mRNA by the NMD machinery activated by Ddx5.

NMD in mammals largely occurs during the pioneer round of translation (62), and in agreement with this fact, we found Ddx5 preferentially interacting with CBP80-bound mRNPs. Immunoprecipitated mRNPs containing eIF4E [and most probably also eIF4G instead of CTIF; (80)] may have escaped degradation in the first round of translation and be recognized by the NMD machinery in following ones as has been shown in the yeast *S.cerevisiae* (81) and recently also in human cells (65,82).

The Ddx5-mediated expression control of Smg5 is of special interest. Smg5 itself is an essential NMD factor, and its mRNA was shown before to be NMD regulated in a feedback regulatory manner (78,83,84). As with other NMD-sensitive transcripts, its long 3′-UTR was identified as the main NMD-inducing feature, although the mechanism that discriminates between NMD-sensitive and -insensitive mRNAs with similarly long 3′-UTRs remained unclear (7,77,78). *Smg5*, like some other wild-type genes, has been reported to undergo NMD regardless of severely reduced Upf3B levels, leading to the notion that an entity other than the classical EJC may serve as a signal for an alternative branch of the NMD pathway (18). In fact, according to our data, Ddx5 seems to take over the role of the EJC in *Smg5* regulation. It has been shown that the expression of Upf3B is regulated in humans (85,86) and, therefore, also may limit Ddx5-mediated NMD in HeLa cells resulting in the downregulation of Smg5 (as well as the Ddx17) levels by FLAG-Upf3B overexpression. This situation may become even more complicated when paralog Upf3A compensates for the loss of Upf3B as reported previously (86). Furthermore, the Ddx5-Upf3 complex may also have different affinities to individual mRNAs, which could explain why Ddx5-mediated NMD of Smg5, in comparison with Ddx17, seems more impervious to Upf3B depletion in the RNAi approach. Competing for the Upf3B function by expression of non-functional mutants (hUpf3B_1__–__270_, not binding Ddx5, and hUpf3B_270__–__470_, not binding hUpf2) verified the hitherto challenged role of Upf3B in Smg5 regulation (18). We further demonstrate that the 3′-UTR of *Smg5, Ddx5* and *Ddx17* mRNA is the distinguishing mark for Ddx5-dependent degradation. Interestingly, the Ddx5 mRNA is missing putative NMD-inducing features like a long 3′-UTR (Figure 6A) or ORFs, confirming previous reports on NMD of physiological mRNAs in yeast and humans (6–11). In contrast, the 3′-UTR of *Tram1* mRNA, irrespective of its length (1494 nt), leaves the reporter construct insensitive to Ddx5-induced NMD (19). Our expression analyses are supported by RIP, showing that mRNPs regulated by Ddx5 are preferentially bound by it *in vivo*. Furthermore, this interaction seems to cover the 3′-UTR as deduced from the RIP experiment performed with the 3′-UTR-Smg5 containing reporter construct. So far, we could not test whether tethering Ddx5 to the 3′-UTR of a reporter results in NMD induction because fusion of the RNA-binding peptide of the bacteriophage λ-anti-terminator protein N (λN-peptide) to the N- and C-terminus of Ddx5 resulted in an extremely low expression level or in a non-Upf3B-binding fusion protein, respectively (unpublished observations). According to our results, Ddx17 also binds to Upf3, which is not unexpected and seems to correspond, e.g. with the regulation and partially redundant function of Upf3 paralogs Upf3A and Upf3B in the PTC-dependent NMD (18). It remains to be determined whether the Ddx17-Upf3 interaction is direct or indirect by complex formation with Ddx5, and whether it can compensate for the loss of Ddx5 by regulating its NMD target transcripts.

As Ddx5 is a nuclear-cytoplasmic shuttle protein required throughout major nuclear steps of gene expression (38), we propose that in complex with Upf3, it becomes part of mRNPs by binding to the 3′-UTR of certain mRNAs in the nucleus. Indeed, preferred binding of Ddx5 to the *Ddx5*, *Ddx17* and *Smg5* mRNA could be demonstrated, and in case of Smg5, this binding could be localized to the 3′-UTR. Nevertheless, it seems to be a critical step, as the Ddx5 ATP-binding mutant, shown before to lack an efficient RNA binding and structure rearrangement activity (32), was not able to induce the NMD process. We propose that after addition of Upf2 to the Ddx5-Upf3 complex in the cytoplasm, the interaction of the eRF1–eRF3 termination complex with PABP, otherwise essential for efficient termination of translation, will be disturbed. As a consequence [and in analogy to the EJC model; see (87)], the NMD core factor Upf1 and its kinase partner Smg1 is recruited by eRF3 into the SURF complex (Smg1–Upf1–eRF1–eRF3). Eventually, a functional NMD complex is formed by contact of Upf1 with NMD core factors Upf2 and Upf3B, triggering Smg1-mediated phosphorylation of Upf1, translation repression and rapid degradation of the transcript (4,88). Finally, the mRNAs analyzed here are most probably not the only ones regulated by Ddx5, the autoregulation of which, like that of some other NMD factors, is NMD controlled in a feedback regulatory manner. Furthermore, it remains to be determined whether the new NMD function of Ddx5 participates in the cell growth regulation [which has also been demonstrated to depend on the ATP-binding activity of Ddx5; (32)] and/or the tumorigenic activity of this multifunctional protein [for a review, see (89)].

## SUPPLEMENTARY DATA

Supplementary Data are available at NAR Online: Supplementary Tables 1–3.

## FUNDING

HOMFOR grant program of the Medical Faculty of Saarland University (Homburg, Germany); Graduate Scholarship Program of the Saarland University (to V.G.). Funding for open access charge: University of Saarland.

*Conflict of interest statement*. None declared.

## Supplementary Material

Supplementary Data
