# Supplementary material for: The RNA helicase Ddx5/p68 binds to hUpf3 and enhances NMD of Ddx17/p72 and Smg5 mRNA
Source: Nucleic Acids Res. 2013 Jun 20;41(16):7875–88. doi: 10.1093/nar/gkt538 (PMC3763533; doi:10.1093/nar/gkt538)
Supplement: Supplementary Data [file supp_41_16_7875__index.html]

The RNA helicase Ddx5/p68 binds to hUpf3 and enhances NMD of Ddx17/p72 and Smg5 mRNA — The RNA helicase Ddx5/p68 binds to hUpf3 and enhances NMD of Ddx17/p72 and Smg5 mRNA — Supplementary Data 

# The RNA helicase Ddx5/p68 binds to hUpf3 and enhances NMD of Ddx17/p72 and Smg5 mRNA

## 

files

**Files in this Data Supplement:**

- Supplementary Data - pdf file
