# Supplementary material for: The RNA helicase Ddx5/p68 binds to hUpf3 and enhances NMD of Ddx17/p72 and Smg5 mRNA
Source: Nucleic Acids Res. 2013 Jun 20;41(16):7875–88. doi: 10.1093/nar/gkt538 (PMC3763533; doi:10.1093/nar/gkt538)
Supplement: Supplementary Data [file supp_gkt538_nar-00461-v-2013-File008.pdf]

Supplementary Tables

Supplementary table 1 Primers used for construction of plasmids

constructs	Forward primer 5´-3	Reverse primer 5´-3
**pCIneo-FLAG-Upf3B 1-270**	CGCGCTCGAGATGAAGGAAGAGAAGGA	CGCGGTCGACTCACAGCTTAATCTT
**pCIneo-FlAG-Upf3B 270-470**	CGCGCTCGAGAAGAAGCAAGAAAAAGGA	CGCGGTCGACTCTAGATCACTCCTCT
**pCMV-Ddx5 1-189 KT3**	GCAGCAAGTAGCTGCTGAACCACCAGAGCCTGAGACAT	ATGTCTCAGGCTCTGGTGGTTCAGCAGCTACTTGCTTGC
**pCMV-Ddx5 190-614 KT3**	TGTCCACTCCGACGCCATGTATTGTAGAGCATGTCGCT	AGCGACATGCTCTACAATACATGGCGTCGGAGTGGACA
**pRTS-hygro-Ddx5-HA**	CGCAGGATATCCCACCATGGCGGGTTATTCG	CGCAGGATATCTTGGGAATATCCTGTTGGC
**pCIneo-EGFP-3´UTR-Ddx17**	CGCGTCCGGATAAAACCACTCAAGTGGTAGTGACTCCAGCACGCGTCCGGATAAAACCACTCAAGTGGTAGTGACTCCAGCA	CGCGGCGGCCGCCAAGATGATGGTATC
**pCIneo-EGFP-3´UTR-Smg5**	GAACTCCGGATGATACTGACCCCCAGGCCCTG	ACCTGCGGCCGCGGTTTATTAAGTGATGCTTTAGTCTCAG
**pCIneo-EGFP-3´UTR-Tram1**	CAACTCCGGATAATGAATTATAAACTAATTGATTAATGTCCCC	ACCTGCGGCCGCGATTGATTTCTCAATGTATAGTTCAGTATA
**pCIneo-EGFP-3´UTR-Ddx5**	CAACTCCGGATAAGACTTTAGAAGTATATGTAAATGTCTG	ACCTGCGGCCGCTCACCTGTATCAATTCATTCTGACTC

Supplementary table 2 siRNAs used

siRNA	Target sequence 5´-3´
**Ddx5si A**	AACCGCAACCATTGACGCCAT
**Ddx5si B**	GAGATTACACCTCACGCTG
**Ddx5+17si**	AAGGCTAGATGTGGAAGATGT
**Upf1si**	AAGATGCAGTTCCGCTCCATT
**Upf2si**	AAGGCTTTTGTCCCAGCCATC
**Upf3si**	AAGGAGAAGCGAGTAACCCTG

Supplementary table 3 Primers used for semi-quantitative RT-PCR

Primers	Forward primer 5´-3´	Reverse primer 5´-3´
**qDdx17**	TCAAGTTTGTGATCAACTATG	ACTACCACTTGAGTGGTTTCA
**qDdx5**	CAAACATGGAAAAGCTCCTATT	CTCTGTCTTCGACCAACTGA
**qSmg5**	GATATCAGAATGAATTAGCTGGCG	CTCACACTTCTTCAGTTGGTGGTAC
**qTram1**	AGGAACAGAAAATGGTGTGAATGG	AACAGGAAAATCTCTAATGCTGAAAG
**qGAPDH**	TGAAGGTCGGAGTCAACGGATTTGGT	GCAGAGATGATGACCCTTTTGGCTC
**qEGFP**	CACCTACGGCAAGCTGA	CATGTGATCGCGCTTCT
**Firefly**	CACTTACGCTGAGTACTTCG	GCAACCCCTTTTTGGAAACG
**Renilla**	TTCGTGGAAACCATGTTGCC	TGGAAAAGAATCCTGGGTCC
